# Risk Factors Associated with Default among New Smear Positive TB Patients Treated Under DOTS in India

**DOI:** 10.1371/journal.pone.0010043

**Published:** 2010-04-06

**Authors:** Sophia Vijay, Prahlad Kumar, Lakbir Singh Chauhan, Balasangameshwara Hanumanthappa Vollepore, Unnikrishnan Pallikkara Kizhakkethil, Sumathi Govinda Rao

**Affiliations:** 1 National Tuberculosis Institute, Bangalore, India; 2 Central Tuberculosis Division, Directorate General of Health Services, Ministry of Health and Family Welfare, New Delhi, India; 3 Independent Consultant, TB Lab Support, Bangalore, India; 4 Deputy Director General, NSSO(FOD), Zonal Office, Bangalore, India; McGill University, Canada

## Abstract

**Background:**

Poor treatment adherence leading to risk of drug resistance, treatment failure, relapse, death and persistent infectiousness remains an impediment to the tuberculosis control programmes. The objective of the study was to identify predictors of default among new smear positive TB patients registered for treatment to suggest possible interventions to set right the problems to sustain and enhance the programme performance.

**Methodology & Principal Findings:**

Twenty districts selected from six states were assigned to six strata formed, considering the geographic, socio-cultural and demographic setup of the area. New smear positive patients registered for treatment in two consecutive quarters during III quarter 2004 to III quarter 2005 formed the retrospective study cohort. Case control analysis was done including defaulted patients as “**cases**” and equal number of age and sex matched patients completing treatment as “**controls**”. The presence and degree of association between default and determinant factors was computed through univariate and multivariate logistic regression analysis. Data collection was through patient interviews using pre-tested semi structured questionnaire and review of treatment related records. Information on a wide range of socio demographic and patient related factors was obtained. Among the 687 defaulted and equal numbers of patients in completed group, 389 and 540 patients respectively were satisfactorily interviewed. In the logistic regression analysis, factors independently associated with default were alcoholism [AOR-1.72 (1.23–2.44)], illiteracy [AOR-1.40 (1.03–1.92)], having other commitments during treatment [AOR-3.22 (1.1–9.09)], inadequate knowledge of TB [AOR-1.88(1.35–2.63)], poor patient provider interaction [AOR-1.72(1.23–2.44)], lack of support from health staff [AOR-1.93(1.41–2.64)], having instances of missed doses [AOR-2.56(1.82–3.57)], side effects to anti TB drugs [AOR-2.55 (1.87–3.47)] and dissatisfaction with services provided [AOR-1.73 (1.14–2.6)].

**Conclusion:**

Majority of risk factors for default were treatment and provider oriented and rectifiable with appropriate interventions, which would help in sustaining the good programme performance.

## Introduction

Poor treatment adherence increasing the risk of drug resistance, treatment failures, relapses, deaths and prolonged infectiousness remains an hurdle to the success of tuberculosis programmes [1], [2]. Countries implementing DOT to ensure treatment adherence have shown impressive results with increasing treatment success and low default rates [3], [4], [5]. The Revised National Tuberculosis Programmes (RNTCP) based on the internationally acclaimed DOTS strategy has made rapid strides since its implementation. The DOTS is now accessible to more than a billion people in India [4]. The overall programme performance, particularly, with regards to high cure and low default rates has been consistent after RNTCP implementation. Nonetheless, more than 30% of the states still report a cure rate of less than 85% and a default rate of more than 5% [6]. At this juncture, achieving and sustaining high cure rates in all the districts is a major challenge.

Essence of DOTS strategy is to provide standardized care to patients suffering from TB in a manner acceptable to them [7]. Directly observed treatment ensuring treatment compliance is one of the important components of the strategy. However, delivery and utilization of DOT services present a wide range of challenges for providers and patients which, presumably depend on the geographical, demographic and socio-cultural diversities in the country.

Treatment success, results from interplay between the programme expectations and the strife of both patients and health care providers to meet them. Certain demands are thus imposed on patients by health providers to fulfill the programme expectations. Patients in turn have multiple and conflicting obligations concerning employment, family and society, which they have to overcome to comply with treatment. Therefore, default is viewed as a product of provider problems and limitations as much as patients. Hence, non adherence to treatment should consider the entire gamut of contributing factors and not restrict only to reasons for default stated by patients [8], [9]. In RNTCP the onus of treatment success lies entirely on the health provider. To prevent defaults, the programme guideline recommends prompt and repeated retrieval actions through home visits for patients missing a dose. Consequently, address verification before treatment initiation becomes mandatory for successful patient retrieval [10].

This study was conducted with the objective of determining risk factors for default encompassing patient and provider related issues in the districts coming under different geographic terrain, socio-cultural milieu and demographic structure. The in-depth evaluation of the delivery of DOT services and its utilization by patients was not only to identify factors influencing treatment adherence but to suggest interventions for rectifying them. Though earlier studies have addressed the issue of treatment default [11]–[13] this study takes into consideration the diversities within the country as well.

## Methods

### Study design, settings

This was an observational study with a ***retrospective cohort of new smear positive (NSP) TB patients aged ≥15 years***. Within the cohort a nested case control study design was used wherein patients defaulting from treatment were considered as ‘*cases*’ and those completing treatment as ‘*controls*’.

**Figure 1:**
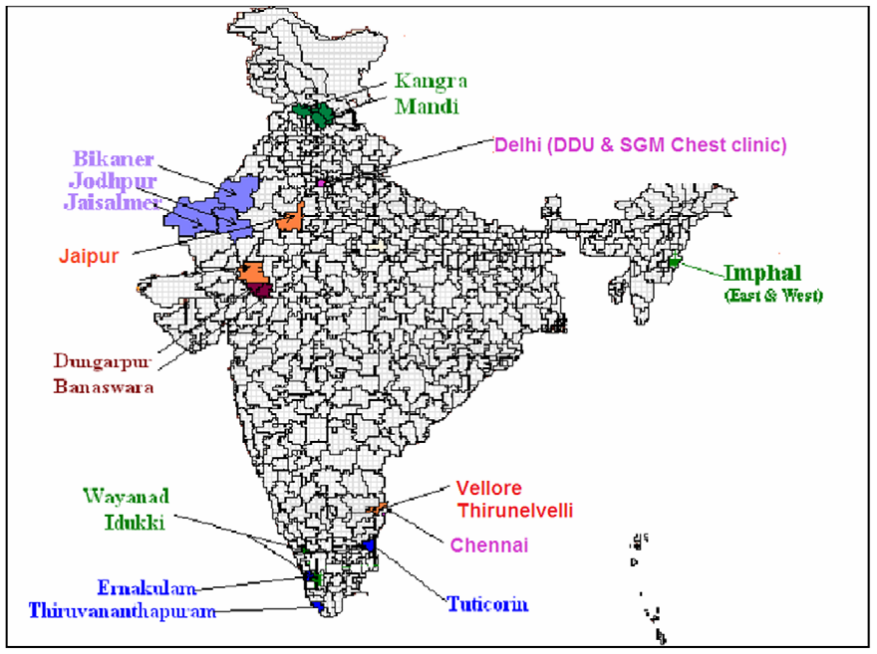
Districts selected for the study. Distribution of districts in each strata: **Hilly** - Kangra, Mandi, Imphal (East and West), Wayanad, Idukki, **Coastal** - Tuticorin, Thiruvananthapuram, Emakulam, Plain - Jaipur, Vellore, Thirunelveli, **Desert** - Bikaner, Jodhpur, Jaisalmer, **Tribal** - Dungarpur, Banaswara, **MC** - Chennai, Delhi (DDU & SGM Chest clinic).

Nine states were selected on the criteria of complete coverage under RNTCP [14]. Considering socio-cultural, demographic and geographical diversities within and between the states, six strata were identified. viz. plain, desert, coastal, hilly, tribal and Municipal Corporation (MC). The districts in these states were subsequently assigned to the above strata. The predominant physical feature was the criterion for designating districts under each geographical stratum – plain, desert, coastal and hilly. MCs of metropolitan cities with different demography, health infrastructure and tribal areas [15] with distinct cultures were considered as separate strata. Within each stratum, districts were listed in a descending order of NSP cases initiated on treatment in a specified quarter. First two to three districts were then selected from the list. Thus, 20 districts selected from six states (Tamilnadu, Kerala, Rajasthan, Himachal Pradesh, Delhi and Manipur) formed the study area (Figure 1).

NSP patients registered for treatment during two consecutive quarters in the selected districts, six months prior to commencement of the field work formed the study cohort. After the declaration of treatment outcome of the cohort, for every defaulted patient (case), one patient from completed group (control) was matched by age group, gender and treatment center. Thus, patients ‘***defaulted***’ from treatment and an equal number of patients from ‘***completed***’ group constituted the ***study group***. These patients had received CAT I regimen (2H_3_R_3_Z_3_E_3_/4H_3_R_3_) as per programme guidelines [10]. In the intensive phase (IP) the drugs were supposed to be administered under direct observation thrice weekly, while in the continuation phase (CP), the first dose of the week was to be given under observation and the rest were self administered. Drugs were dispensed in patient-wise boxes each containing blister packs for daily and weekly administration in IP and CP respectively. DOT was provided either at the health center/sub-center or at community level by a designated DOT provider.

### Definitions and data collection

***Defaulted*** was defined as patients interrupting treatment for more than eight weeks consecutively after initiation of treatment [10]. ***Completed*** were patients completing the prescribed course of treatment irrespective of final sputum result. Patients were considered to have ***Instance of missed dose*** when they failed to consume a dose within the stipulated interval of two days in IP or one collection within a week in CP.

Data was collected through patient interviews using pre-tested questionnaire and review of TB registers & treatment cards. Enrolled patients were traced and interviewed by trained field investigators. Interviews were conducted in regional language 2–4 months after the declaration of treatment outcome by the field investigators in the presence of a field supervisor. The questionnaires were checked for consistency and correctness by the field supervisors on completion of interview. Canvassing of the interview schedule was standardized by practical exercises and field training of the staff. Prior to interviews, formal introduction was given to patients regarding the purpose of seeking information and informed oral consent was obtained. The questionnaire had built-in checks to ensure reliability and consistency of information obtained. Specific guidelines were provided for filling up the questionnaire to avoid ambiguity. To ensure adequate coverage minimum three attempts were made to trace each patient.

The questionnaire had two parts - *patient profile and treatment details*. Patient profile covered the socio-demographic data, knowledge and perception about TB, personal experience and practices adopted during treatment period by patients'. Information on habits and other associated illness was also sought. Support received from family, relatives and the health staff during the treatment which could have influenced patient's treatment compliance was also obtained. Under treatment details, particulars regarding DOT like initial home visit by health staff, place of DOT, regularity of treatment, side effects to drugs and action taken for the same were collected from the patient. A set of questions focused on adequacy of patient provider interaction. Patient's opinion about DOT and satisfaction with the services provided at the government health center was also obtained.

The relevant treatment details of each patient like treatment center, place of DOT, regularity of drug consumption in IP/CP, instances of missed dose and treatment outcome were recorded in the interview schedule from the treatment card which also facilitated interviews. Pilot testing of study procedures and questionnaire was conducted in two districts of Karnataka state and questionnaire was modified suitably [16]. Field work for data collection was carried out in three phases from July 2005 to December 2006.

### Data Analysis

Data management and analysis was done using FoxPro 6.5 and SPSS-version 10. Double data entry procedure was adopted and digitized data were checked for completeness and consistency. The association of potential socio-demographic and treatment related risk factors among defaulted and completed group was initially studied through univariate analysis within and between the stratum. The categorical variables were assessed using Pearson chi-square. Mantel Hanzel Odds Ratio (OR) and corresponding 95% Confidence Interval (CI) were calculated for dichotomous variables.

Logistic regression analysis was then undertaken to estimate the independent effect of the factors that were significantly associated with default. Variables yielding p values <0.1 in univariate analysis were included in the logistic regression model. A backward stepwise elimination procedure based on the likelihood statistics, (using probability of 0.1 for removal and 0.05 for entry) was also performed to identify the best subset of variables as risk factors. Statistical tests were carried out at 5% level of significance.

### Ethical consideration

The purpose for seeking information was explained in detail to individual patient. Only informed verbal consent was obtained prior to interview as there was no other intervention/procedure involved. Patients' consent to participate in the interview was recorded on the individual interview schedule which was signed by the interviewer. The data collected was presented as an aggregate and was not linked to any individual in the study. Patients were assured that non participation in the interview will not jeopardize their access to any government health center subsequently. As a service component, patients were informed about the disease and its treatment to bridge the observed gap in their knowledge and defaulted patients were motivated to resume treatment. The data obtained from patient records and interviews were securely held and confidentially maintained by study staff. The research activity was approved by the Institution Ethics Committee of National Tuberculosis Institute. Approval for the research was also accorded by the Ministry of Health and Family Welfare, Government of India.

## Results

**Figure 2:**
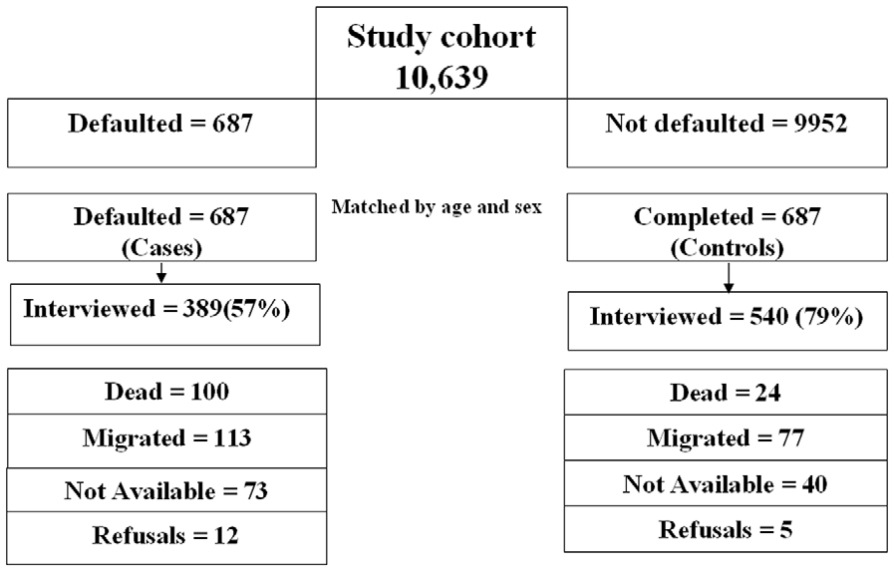
Interview coverage of the study group.

The study cohort comprised 10,639 NSP patients aged >15 years from two consecutive quarters during III quarter 2004 to III quarter 2005. In the cohort, 687 (6.4%) ‘Defaulted’ and equal number of patients from the ‘completed’ group constituted the study group. Of these, 389 (57%) in the defaulted and 540 (79%) in the completed group could be interviewed (Figure 2).

### Personal and Socio-demographic risk factors

Univariate analysis of these factors irrespective of strata revealed the significant association of default with illiteracy, patients having other commitments during treatment period, Alcoholism and smoking (Table 1).

**Table 1:** Overall univariate analysis of association of socio-demographic and treatment related factors with treatment default among new smear positive TB patients.

Factors	Def N-389	Comp N-540	P value	OR (95% C.I)
**Patient related**				
<Median age (41 yrs)	195	268	0.88	1.020 (0.79–1.32)
Resident for <1year	21	31	0.82	0.94 (0.53–1.66)
Literate (Read & write)	208	339	0.004	0.68 (0.52–0.89)
Married	303	433	0.39	0.87 (0.63–1.19)
Employed	216	306	0.73	0.95 (0.73–1.24)
Patient sole earner	92	130	0.88	0.97 (0.72–1.33)
Had other commitments	96	64	0.00	2.44 (1.72–3.45)
Alcoholic	191	180	0.00	1.93 (1.48–2.52)
Smoker	205	232	0.003	1.48 (1.14–1.92)
Having nuclear Family	255	349	0.77	1.04 (0.79–1.37)
**Treatment related**				
Poor knowledge of TB and treatment	156	125	0.000	2.22 (1.70–3.00)
Had associated illness	57	99	0.14	0.77(0.54–1.09)
Inadequate patient provider interaction	298	335	0.000	2.00 (1.48–2.71)
Address verification not done	257	320	0.03	1.34 (1.01–1.77)
DOT at Health center/sub Center	319	456	0.32	0.84 (0.59–1.19)
Distance to DOT center (< = 2km)	309	437	0.57	0.91(0.66–1.26)
Patient with instances of missed doses	207	425	0.000	3.25 (2.42–4.37)
DOT not done	170	197	0.03	1.35 (1.03–1.80)
Side effects to drugs	212	149	0.000	3.14 (2.39–4.14)
*Overlapping of working hours with DOT timing	52	78	0.71	0.93 (0.61–1.42)
*Out station duties during treatment	18	12	0.033	2.23 (1.05–4.72)
Family support	377	530	0.22	0.59 (0.23–1.49)
Poor support from health staff	34	6	0.000	8.52 (3.40–22.83)
Unsatisfied with services	72	10	0.000	12.04 (5.92–25.20)

*Only for employed patients.

Def – Defaulted, Comp-Completed, OR – Odds Ratio, CI – Confidence Interval.

Strata wise univariate analysis showed no significant differences between defaulted and completed groups for any socio-demographic factors in the hilly and desert strata. **Literacy rate** [17] was significantly lower among patients in defaulted in comparison with completed group in plain (p = 0.01) and MC strata (p = 0.03). Though alcoholics were higher among defaulted in all the strata, the association of **alcoholism** with default was significant only in coastal [OR-3.2,CI (1.5–6.8)], plain [OR -1.8,CI(1.1–2.9)] and MC [OR-2.3,CI(1.3–4.2)] strata. **Important commitments** like weddings / functions, festivals, work etc., during treatment period was also associated with default among patients in coastal [OR- 3.2,CI(1.0–10.2)], plain [OR-2.1,CI(1.2–3.9)], tribal [OR-3.8,CI(1.5–9.4)] and MC [OR-4.6,CI(2.2–9.4)] strata. Association of **Smoking** with default was observed in coastal [OR-3.1, CI(1.4–6.6)] and MC [OR-2.0,CI(1.1–3.6)] strata. Proportion of patients **employed** and having **nuclear families** though not significant were higher among the completed group in all the strata. (Table 2)

**Table 2:** Strata-wise univariate analysis of association of patient related socio demographic factors with treatment default among new smear positive TB patients.

Factors	Strata																	
	Hilly			Coastal			Plain			Desert			Tribal			MC		
	*Def N = 31	*Comp N = 50	P value	Def N = 48	Comp N = 74	P value	Def N = 126	Comp N = 159	P value	Def N = 53	Comp N = 69	P value	Def N = 51	Comp N = 67	P value	Def N = 80	Comp N = 121	P value
**>Median age (41 yrs)**	16	19	0.22	15	25	0.77	54	71	0.76	29	37	0.9	34	52	0.18	47	64	0.41
**Resident for >1 year**	1	1	1	2	6	0.47	8	6	0.31	0	1	0.37	51	67	-	10	17	0.75
**Read & write**	20	35	0.6	41	62	0.8	64	106	**0.01**	18	24	0.92	19	25	1	46	87	**0.03**
**Married**	22	42	0.16	30	63	**0.00**	111	135	0.43	44	54	0.51	41	53	0.86	55	86	0.72
**Living in nuclear family**	25	34	0.2	33	48	0.65	87	102	0.39	21	29	0.89	29	44	0.33	60	92	0.87
**Employed**	17	34	0.23	26	42	0.77	58	83	0.3	25	29	0.57	39	40	0.06	51	78	0.92
**Patient sole earner**	10	18	0.73	12	16	0.66	19	27	0.66	16	13	0.14	18	15	0.12	17	41	0.05
**Had other commitments**	0	0	-	9	5	**0.04**	32	22	**0.01**	6	14	0.18	19	9	**0.00**	30	14	**0.00**
**Alcoholic**	14	21	0.78	29	24	**0.00**	64	58	**0.01**	14	9	0.06	25	25	0.2	45	43	**0.00**
**Smoker**	16	27	0.83	32	29	**0.00**	76	81	0.11	15	21	0.79	22	33	0.86	42	43	**0.02**

*Def – Defaulted, Comp - Completed.

### Treatment related risk factors

In the overall univariate analysis, factors significantly associated with default were, poor knowledge regarding TB and its treatment, inadequate patient provider interaction, address verification not done, patients with instances of missed doses, DOT not done, side effects to TB drugs, frequent outstation duties during treatment period, poor support from health staff and dissatisfaction with services provided. (Table 1)

Strata wise Univariate analysis of treatment related factors is given in Table 3. ***Patients Knowledge*** regarding susceptibility to TB, its cause and spread, treatment duration, regularity and curability was assessed through a set of questions. Defaulted patients in all the strata exhibited poor knowledge compared to the completed group and the difference between the two groups was significant in coastal (p = 0.02), plain (p<0.01) and MC (p = 0.01) strata. ***Poor patient provider interaction*** was a potential risk factor for default in hilly [OR-3.77,C.I(1.12–13.40)], tribal [OR-2.96,C.I(1.30–2.82)] and MC [OR-2.43,C.I(1.23–4.83)] strata. ***Initial home visit for address verification*** though not done in 62% of patients in the study group, was not associated with default in any of the strata. Proportion of ***patients with instances of missed doses*** during the treatment was significantly higher among defaulted than completed group in all the strata except desert and tribal. ***DOT not done*** during IP did not show any association with default of the strata. The proportion of patients' receiving DOT irrespective of defaulted or completed was least (41%) in plain and was significantly higher in MC (86%) and coastal (79%) compared to other strata. **Side effects** to anti-TB drugs were reported by higher proportion of patients in defaulted than completed group in all the strata except MC and tribal. Patients with frequent **outstation duties** during the treatment period were also significantly higher (p = 0.03) in defaulted of MC strata. However, this factor was not included for multivariate analysis as outstation duties were considered only for employed patients. Majority (>80%) of the patients in both defaulted and completed group in all the strata expressed getting ***support and co-operation from the health staff***. Nonetheless, a higher proportion of patients in defaulted group reported poor staff support in hilly (p = 0.03), plain (p = 0.001) & MC (p = 0.01) strata compared to completed group. Though 847 of the 929 (91%) of the patients in the study group expressed ***Satisfaction with the DOT services***, there was significant difference in this regard between the defaulted and completed group (81% v/s 98%, p<0.01). The difference between the two groups was also significant in all the strata except coastal.

**Table 3:** Strata-wise univariate analysis of association of treatment related factors with treatment default in new smear positive TB patients.

Factors	Strata																	
	Hilly			Coastal			Plain			Desert			Tribal			MC		
	*Def N = 31	*Comp N = 50	P value	Def N = 48	Comp N = 74	P value	Def N = 126	Comp N = 159	P value	Def N = 53	Comp N = 69	P value	Def N = 51	Comp N = 67	P value	Def N = 80	Comp N = 121	P value
**Knowledge of TB**	28	44	0.75	23	51	**0.02**	64	122	**0.00**	30	48	0.14	40	56	0.48	48	94	**0.01**
**Had associated illness**	5	7	0.8	8	24	0.05	22	25	0.69	6	6	0.63	3	2	0.65	13	35	**0.03**
**Adequate patient provider interaction**	5	21	**0.01**	26	45	0.47	14	30	0.07	11	19	0.39	17	40	**0.00**	18	50	**0.01**
**Address verification**	8	22	**0.1**	23	37	0.82	33	57	0.08	16	23	0.71	20	38	0.06	32	43	0.52
**DOT at health centre**	20	37	0.36	26	34	0.37	102	143	**0.03**	49	66	0.45	46	62	0.65	67	112	0.5
**Patients with instances of missed doses**	11	3	**0.00**	28	15	**0.00**	65	40	**0.00**	13	12	0.33	21	19	0.15	44	27	**0.00**
**DOT done**	19	33	0.67	38	58	0.92	47	71	0.21	23	34	0.52	27	40	0.46	65	107	0.16
**Side effects to drugs**	17	12	0.01	20	6	**0.00**	81	54	**0.00**	33	15	**0.00**	28	25	0.06	33	37	0.12
**Outstation duties uring treatment**	0	0	-	0	1	1	6	5	0.35	0	2	0.49	5	2	0.26	7	2	**0.03**
**Overlapping of working hours with DOT timing**	16	29	0.65	21	36	0.6	43	63	0.81	18	23	0.53	29	33	0.38	37	44	0.06
**Family support**	29	48	0.62	47	74	0.39	109	155	0.22	53	68	1	51	66	1	78	119	0.65
**Staff support**	26	49	**0.03**	47	74	0.39	115	158	**0.00**	47	67	0.08	49	66	0.58	73	120	**0.01**
**Satisfied with services**	23	49	**0.00**	47	73	0.76	100	159	**0.00**	38	64	**0.00**	44	65	**0.03**	65	120	**0.00**

*Def – Defaulted, Comp - Completed.

In the overall logistic regression analysis, the socio-demographic and treatment related factors independently associated with default were, alcoholism [aOR-1.72], illiteracy [aOR-1.40], having other commitments during treatment [aOR-3.22], inadequate knowledge of TB [aOR-1.88], inadequate patient provider interaction [aOR-1.72], no support from health staff [aOR-1.93], instances of missed doses [aOR-2.56], side effects to anti-TB drugs [aOR-2.55] and dissatisfied with services [aOR-1.73] (Table 4). The overall prediction of default through the logistic regression for the observation was 73%. Analysis of risk factors independently associated with default in individual strata is given in Table S1

**Table 4:** Overall multivariate analysis of socio-demographic and treatment related risk factors and their association with default.

Variables	aOR	95% CI	p value
**Alcoholism**			
Alcoholic/non alcoholic	1.72	1.23–2.44	.002
**Smoking**			
Smoker/non smoker	1.12	0.77–1.64	.552
**Literacy**			
not able to read & write/able to	1.408	1.03–1.92	.032
**Other commitments**			
Had/did not have	3.22	1.12–9.09	.030
**Knowledge of TB**			
inadequate/adequate	1.88	1.35–2.63	.000
**Address verification**			
Not done/done	1.37	1–1.88	.053
**Patient provider interaction**			
Inadequate/adequate	1.72	1.23–2.44	.002
**Health staff support**			
Inadequate / adequate	1.93	1.41–2.64	.000
**DOT**			
Not done /done	1.01	0.73–1.39	.925
**Instances of Missed doses**			
Had missed dose / No missed dose	2.56	1.82–3.57	.000
**Side effect**			
Had/did not have	2.55	1.87–3.47	.000
**Satisfaction with services**			
Not satisfied / satisfied	1.73	1.14–2.6	.009
**Prediction %**	73%		

aOR – Adjusted Odds Ratio, CI – Confidence Interval.

## Discussion

The question often arises why some patients complete treatment successfully while others don't under comparable conditions. Seeking answer in this direction, a comparison was made between the defaulted and completed group of TB patients regarding the personal, socio-demographic and logistic aspects, using a nested case control study design. Despite the retrospective study design interview coverage of 68% (57% in defaulted and 79% in completed group) was achieved. A comparatively lower coverage of defaulted group was mainly due to higher deaths (15%) compared to those in the completed group (4%). Higher deaths among defaulted was probably due to irregular and inadequate treatment as evident from patients missing doses (48%) and stopping treatment in IP (62%).

Logistic regression analysis in search for factors strongly associated with default, revealed alcoholism as a risk factor overall and particularly in the coastal and MC strata. Alcoholism has been identified as an important predictor of noncompliance in several studies in different parts of the world [11], [12], [18]–[22]. Elicitation of history of alcoholism prior to treatment initiation will help in identifying potential defaulters needing special attention during treatment. Improving compliance among alcoholic patients through support from family, health staff and social organizations is a challenge to be addressed. Side effects to anti-TB drugs, as reported in other studies, [23]–[25] was also strongly associated with default in all the strata except MC and tribal. One of the reasons for higher incidence of perceived side effects perhaps could be the continued practice of giving medication on empty stomach in some districts. The DOT providers need adequate orientation regarding possible side effects and prompt referral of patients to the medical officer for remedial measures. Frequently reported minor side effects could be successfully dealt with proper instructions on drug consumption, reassurance to patients and prompt symptomatic treatment before it leads to default.

Effective patient provider interaction is a means of providing treatment related information particularly the importance of DOT and clearing the doubts regarding disease and treatment. This plays a decisive role in enhancing treatment compliance. Poor patient provider interaction has also been reported as a risk factor in studies elsewhere [13], [24], [26]–[28]. Besides, many a times unwarranted information like, necessity of nutritious diet, need for hospitalization, isolation of patient, consuming drugs on empty stomach is conveyed to the patient. Limited interaction, variability in the content of message delivered, delegating responsibility of motivating patients to untrained health staff are the problems identified in poor patient provider interaction [26]. Inadequate and improper patient provider interaction often stems from the lack of time and knowledge of providers themselves. [28].

It is encouraging to note that more than 80% of patients who underwent treatment, had adequate knowledge about TB and its treatment. However, inadequate knowledge emerged as a risk factor for default. The probable reason for this could be lack of patient provider interaction as evident among 89% and 78% of defaulted patients in MC and plain strata respectively. The other contributing factor for inadequate knowledge could be the low literacy rate among defaulted patients of plain and MC strata limiting their access to various information sources [24], [25], [29]–[33]. This emphasizes the need for improving the interpersonal communication skills of the health providers enabling them to impart knowledge regarding TB and its treatment to the patients in tune with their literacy level.

Important commitments during treatment was a hurdle, leading to default. Majority of the patients had stated occupation (40%), festival and functions (39%) as the commitments (not on table). The DOTS programme is rigid in terms of thrice weekly drug administration under direct observation during IP [10]. To fulfill the requisites of DOT, the patient often has to compromise with his personal, family and social obligations which at times becomes difficult to sustain and leads to treatment interruption. Solution to this problem is often difficult. Nevertheless, under compelling circumstances, flexibility could be exercised by providing one or two blister packs in IP for self administration with the knowledge of the medical officer and its documentation on the treatment card. This would further strengthen the confidence of patients in the system. But, such situations should be dealt with extreme caution and rather be exceptions than norm. Poor support from the health staff during treatment was a significant predictor for default. Support and co-operation to the patient from care providers by providing counseling, building good rapport, insistence on treatment regularity, repeated motivation, empathetic attitude, timely provision of drugs, are important in ensuring treatment regularity.

The fact that only 61% of the patients in the study group received DOT and that the proportion was significantly lower among defaulted than completed group (56% v/s 63%) is a matter of concern. DOT seemed to be more organized in districts of MC and coastal strata. Evidence and experience shows that treatment adherence through DOT, particularly, with intermittent regimens is an important determinant of the overall success or failure of the programme [31].

Dissatisfaction with treatment services and poor patient provider interaction were apparent determinants of default and barriers in utilization of services [33], [34]. Reasons for dissatisfaction were lack of personal attention, rude behaviour of the staff, inconvenient DOT timings and long waiting hours stated particularly by defaulted patients of plain, desert and MC strata (not tabulated). Managerial staff could deal with these problems by inculcating commitment and motivation among the staff through periodic review of the constraints faced by them, like multiple responsibilities, overcrowding of patients etc. Decentralizing DOT services could also be a measure to circumvent these problems as the study revealed that majority of patients (>60%) in the plain, desert and MC strata received DOT at the primary health center itself. Decentralization of treatment associated with treatment success has been demonstrated in studies in Kenya and Malawi [31], [35], [36].

The possibility of predicting default considering all the risk factors was particularly high in hilly (79%) and coastal (78%) strata (Table S1). It is obvious that predicting default at treatment initiation though difficult the risk factors would help in identifying the potential defaulters requiring attention and repeated motivation. The retrospective study design has enabled to collect information on the treatment related events such as side effects, DOT done, patient provider interaction, support from health care providers etc., which would influence patients decision to continue treatment. These factors not only have a higher prediction but are easily rectifiable with minimum intervention as they are basically provider oriented.

### Limitation

Recall bias which is one of the intrinsic limitations of any retrospective study was minimized by interviewing patients within two to four months of treatment outcome. Two months, though an accepted period for sociological interviews, some of the treatment related details could have been missed due to recall bias. The information lost due to recall was made up to certain extent by collecting the same from treatment related records.

### Conclusion

The study provides an insight into the various issues involved in delivery of DOT services and its utilization for maintaining treatment adherence to achieve the desired cure rate while keeping in view the regional diversities in the country. Some predictors of default were area specific and related to provider and treatment services. The results also endorse the fact that direct enquiry of reasons from patients, may not always yield the true cause for default which is multifactorial. Resorting to indirect methods like comparing factors between defaulted and completed patients may highlight the actual problems not forthcoming through direct questioning.

## Supporting Information

Table S1 Strata-wise multivariate Analysis of Association and Risk Factors for treatment default.
(0.10 MB DOC)
