# Supplementary material for: Risk Factors Associated with Default among New Smear Positive TB Patients Treated Under DOTS in India
Source: PLoS One. 2010 Apr 6;5(4):e10043. doi: 10.1371/journal.pone.0010043 (PMC2850369; doi:10.1371/journal.pone.0010043)
Supplement: Table S1 — Strata-wise multivariate Analysis of Association and Risk Factors for treatment default. (0.10 MB DOC) [file pone.0010043.s001.doc]

**Table S1 - Strata wise multivariate Analysis of Association and Risk Factors for treatment default**

| **HILLY** | | | |  | **DESERT** | | | |
| --- | --- | --- | --- | --- | --- | --- | --- | --- |
| **Risk factors** | **aOR** | **95% CI** | **p value** |  | **Risk factors** | **aOR** | **95% CI** | **p value** |
| Inadequate patient provider interaction | 3.06 | 0.86-1.16 | 0.08 |  | Alcoholic | 2.47 | 0.88-6.94 | 0.09 |
| Instance of Missed doses | 7.813 | 1.82-33.33 | 0.01 |  | Side effects to drugs | 5.07 | 2.21-11.65 | 0 |
| Side effects to drugs | 1.887 | 0.56-6.32 | 0.30 |  | Poor health staff support | 1.41 | 0.19-10.41 | 0.73 |
| Poor health staff support | 4.35 | 0.32-58.82 | 0.27 |  | Unsatisfied with services | 4.76 | 1.45-1.54 | 0.01 |
| Unsatisfied with services | 12.5 | 1.4-111.11 | 0.02 |  |  |  |  |  |
| **Prediction %** | **79%** | | |  | **Prediction %** | **75%** | | |
| **COASTAL** | | | |  | **TRIBAL** | | | |
| Unmarried | 4.09 | 1.43-11.6 | 0.01 |  | Unemployed | 3.09 | 1.11-8.4 | 0.03 |
| Alcoholic | 4.394 | 1.64-0 | 0.00 |  | Other commitments | 4.235 | 1.48-12.09 | 0.01 |
| Smoker | 1.078 | 0.27-4.28 | 0.92 |  | Address verification not done | 1.81 | 0.71-1.81 | 0.21 |
| Other commitments | 2.638 | 0.64-10.92 | 0.18 |  | Distance to DOT centre(>2kms) | 2.55 | 0.93-6.99 | 0.07 |
| Shifted residence | 6.240 | 0.48-81.82 | 0.16 |  | Inadequate patient provider interaction | 4.67 | 1.89-11.49 | 0.00 |
| Inadeqaute knowledge on TB | 2.36 | 0.85-6.58 | 0.10 |  | Side effects to drugs | 2.198 | 0.86-5.61 | 0.10 |
| Associated illness | 1.56 | 0.45-5.46 | 0.49 |  | Not satisfied with services | 10 | 1.44-71.43 | 0.02 |
| Instances of Missed doses | 3.968 | 1.58-10 | 0.00 |  |  |  |  |  |
| Side effects to drugs | 8.425 | 2.60-0 | 0.00 |  |
| **Prediction %** | **78%** | | |  | **Prediction %** | **70%** | | |
| **PLAIN** | | | |  | **MC** | | | |
| Illiterate | 1.61 | 0.88-2.91 | 0.12 |  | Illiterate | 1.67 | 0.77-3.63 | 0.19 |
| Alcoholic | 1.606 | 0.89-2.90 | 0.12 |  | Alcoholic | 2.498 | 1.23-5.06 | 0.01 |
| Other commitments | 2.159 | 1.02-4.55 | 0.04 |  | Smoker | 1.317 | 0.50-3.50 | 0.58 |
| Inadeqaute knowledge on TB | 2.49 | 1.35-4.59 | 0.00 |  | Other commitments | 2.218 | 0.86-5.70 | 0.10 |
| Address Verification not done | 2.89 | 1.48-5.68 | 0.00 |  | Patient sole earner | .487 | 0.21-1.12 | 0.09 |
| Inadequate patient Provider interaction | 1.47 | 0.67-3.22 | 0.34 |  | Associated illness | 4.13 | 1.56-10.99 | 0.00 |
| Place of DOT (Health Center /Sub center) | 0.634 | 0.271-1.48 | 0.29 |  | Inadeqaute knowledge on TB | 2.59 | 1.21-5.52 | 0.02 |
| Instances of missed doses | 2.32 | 1.26-4.27 | 0.01 |  | Inadequate patient Provider interaction | 3.01 | 1.36-6.66 | 0.01 |
| Side effects | 3.356 | 1.86-6.05 | 0.00 |  | Instances of missed doses | 4.065 | 1.99-8.33 | 0.00 |
| Poor health staff support | 12.99 | 0.84-200 | 0.07 |  | Poor health Staff Support | 4.35 | 0.29-62.5 | 0.29 |
| Satisfied with services | 0 | 0 | 0.63 |  | Not satisfied with services | 58.82 | 6.41-500 | .000 |
| **Prediction %** | **74%** | | |  | **Prediction %** | **76%** | | |
